# Comparison of the ability of resection versus nonresection surgery to prevent the recurrence of sigmoid volvulus: A protocol of a meta-analysis and systematic review

**DOI:** 10.1371/journal.pone.0310402

**Published:** 2024-09-24

**Authors:** Xiaomei Jiang, Qiang Du, Lie Yang

**Affiliations:** 1 Division of Gastrointestinal Surgery, Department of General Surgery, West China Hospital, Sichuan University, Chengdu, China; 2 State Key Laboratory of Biotherapy and Cancer Center, Institute of Digestive Surgery, Sichuan University West China Hospital, Chengdu, Sichuan, China; Morgagni-Pierantoni Hospital, ITALY

## Abstract

**Purpose:**

Based on clinical research guidelines and clinical practice, patients with sigmoid volvulus (SV) who receive conservative treatment have a greater recurrence rate than patients who do not receive conservative treatment, which is almost without any controversy. Surgical treatment is usually the final treatment for patients with SV. However, there are multiple surgical methods for the treatment of SV, which can be roughly divided into resection and nonresection methods. The available evidence on the effectiveness of surgery for preventing postoperative recurrence is still inadequate. Therefore, we drafted this systematic review protocol with meta-analysis aimed to compare the effects of these two major types of surgery on preventing the recurrence of SV.

**Methods:**

We comprehensively and systematically reviewed the PubMed, EMBASE, MEDLINE and Cochrane Library databases of articles on SV from inception to November 16, 2023. Two independent authors will screen and analyze the detected literature, and disputes will be resolved through communication with a third experienced person. After evaluating the quality of the literature and estimating the risk of bias, we calculate the pooled effect size and 95% confidence interval. Heterogeneity is analyzed by subgroup analysis, and sensitivity analysis can be carried out to assure the reliability of the results. Finally, the Grading of Recommendations Assessment, Development and Evaluation (GRADE) will be used to evaluate the strength of the evidence. The results of each analysis will be recorded in detail. The whole process was carried out in strict accordance with the Preferred Reporting Items for Systematic Reviews and Meta-Analyses Protocols guidelines (PRISMA-P).

**Trial registration:**

**Protocol registration:** The study protocol has been registered at the International Prospective Register of Systematic Reviews platform (PROSPERO) (CRD42024508350). Protocol version 1.0, 13 Feb 2024.

## Introduction

### Rationale

With 5% of the etiology of large bowel obstruction in developed countries, SV, the most common cause of colonic volvulus, is an emergency disease with high morbidity and mortality [1, 2]. It generally refers to the twist of the intestine and mesentery around the long axis of the intestine [3]. At present, for the treatment of SV, when the vital signs of patients are stable, the main diagnosis and treatment plan is selective surgery after endoscopic decompression [4]. However, patients with intestinal perforation, necrosis or endoscopic decompression failure should be treated with emergency surgery [5]. Endoscopic treatment can only be used as a temporary treatment measure, and patients need additional intervention [6]. Prophylactic surgical treatment after successful decompression, especially during the same admission, is widely recognized and accepted due to the extremely high recurrence rate of endoscopic decompression [7, 8].

There are multiple surgical methods for the treatment of SV, which can be roughly divided into resection and nonresection methods. Nonresection surgery mainly includes sigmoidopexy, Mesosigmoplasty, exteriorization, enterostomy and simple detorsion. The most frequently performed resection surgeries by surgeons are primary resection and anastomosis, Hartmann’s operation, the Paul Miculicz’s operation, and subtotal or total resection [9, 10]. Despite guidelines recommending that final resection management plays a superior role in preventing SV recurrence compared to non-resection surgeries, the insufficient strength of the evidence creates an information gap in SV standard treatment strategies [11].

In conclusion, there is still controversy about which operation has more advantages in preventing recurrence. Therefore, the final conclusion about whether to choose resection treatment has been in the process of discussion.

### Objectives

The main purpose of this meta-analysis is to systematically compare the ability of resection and nonresection surgery to prevent recurrence in patients older than 18 years with SV (confirmed by imaging or laparotomy). The main outcome measure is the postoperative recurrence rate. Postoperative complications are the secondary outcome. Our goal is to establish a framework for surgical treatment to prevent the recurrence of SV and to provide the basis for patients’ individualized diagnosis and treatment decisions through the analysis and elaboration of the relevant surgical value. The planned duration of the study was 1 year, from December 17, 2023, to December 16, 2024.

### Ethics and dissemination

Since this is a study based on published results and does not involve patient intervention, ethical review is not needed. The results of this study will be published in a journal that allows peer review.

## Materials and methods

### The eligibility criteria

This meta-analysis protocol, when fully completed, will be reported strictly in accordance with the Preferred Reporting Items for Systematic Reviews and Meta-Analyses Protocols guidelines (PRISMA-P). The following Outlines the methodology for reporting according to PRISMA-P (S1 Checklist) [12, 13]. This study has been registered on the International Prospective Register of Systematic Reviews platform (PROSPERO). (Registration number: CRD42024508350) [14].

### Study designs

We will include randomized controlled studies and nonrandomized controlled studies, while studies unrelated to surgical treatment, those with no full text, and those with fewer than 10 surgical cases, case reports, reviews, comments, letters, book chapters or conference abstracts will be excluded.

#### Participants

The patients we can choose are 18 years old and older with SV confirmed by imaging or laparotomy. Patients who died or relapsed without surgical treatment are excluded. For studies in which both children and adults are reported at the same time, we selected only adult data if they are expressed separately.

#### Interventions

The intervention measures are resection surgery. Traditional laparotomy procedures include simple open decompression, mesosigmoidoplasty, sigmoidopexy, one-stage resection and anastomosis, the Hartmann operation, and the Paul miculicz operation. New surgical methods, such as percutaneous endoscopic sigmoid colostomy and laparoscopic or laparoscopic-assisted SV surgery, should also be used.

#### Outcomes

The primary outcome of interest is the postoperative recurrence rate. Postoperative recurrence can be divided into two types: early and late. The former is considered to be the number of relapses from the end of surgery to the patient’s current discharge, while late recurrence refers to the recurrence rate from the patient’s departure to the last follow-up. The recurrence rate will be tracked through active patient reporting and electronic medical records systems. If the data for reoperation and endoscopic decompression are not reported, they can be chosen as surrogate outcomes.

If complete data are available, the length of postoperative hospitalization, postoperative complications, postoperative mortality, additional interventions and quality of life within the follow-up period after the operation can be considered secondary outcomes. The Clavien–Dindo grade is used to semi quantitatively describe the severity of postoperative complications [15]. Additional intervention measures refer to surgical failures or complications that require medication or surgical treatment for improvement. In addition, we used the Health Quality of Life Questionnaire (QWB-SA) to evaluate the quality of life of the patients. Pooled quality-of-life statistics were calculated as the mean differences ± standard deviations [16].

#### Setting

There will be no restrictions by type of setting.

### Information sources

We systematically searched PubMed, EMBASE, Medline and the Cochrane Library for studies related to the surgical treatment of SV using medical subject headings (MeSH) and text words from inception to November 16, 2023. The literature search will be limited to the English language and human subjects.

To ensure the comprehensiveness of the search results, we did not impose restrictions on the search terms or screen the references of the included literature.

### Search strategy

The retrieval strategy is improved by the combination of subject words and free words. The core vocabulary mainly included "sigmoid volvulus", "surgery", "management", and "treatment". The corresponding subject words and free words in each database were identified and connected with "or". Next, "management" and "treatment" are combined with "or", and finally, the logical operator "and" is combined with "sigmoid volvulus" and "surgery" to form a search strategy. The search scope was full text. The search formulas are as follows:

PubMed search

#1 General Surgery

#2 (Surgery, General) OR (Surgery)

#3 "Therapeutics"[Mesh]

#4 ((((Therapeutic) OR (Therapy)) OR (Therapies)) OR (Treatment)) OR (Treatments)

#5 "Disease Management"[Mesh]

#6 ((Disease Management) OR (Management, Disease)) OR (Management, Disease)

#7 ((((("General Surgery"[Mesh]) OR ((Surgery, General) OR (Surgery))) OR ("Therapeutics"[Mesh])) OR (((((Therapeutic) OR (Therapy)) OR (Therapies)) OR (Treatment)) OR (Treatments))) OR ("Disease Management"[Mesh])) OR (((Disease Management) OR (Management, Disease)) OR (Management, Disease))

#8 Sigmoid volvulus

#9 (sigmoid volvulus) AND (((((("General Surgery"[Mesh]) OR ((Surgery, General) OR (Surgery))) OR ("Therapeutics"[Mesh])) OR (((((Therapeutic) OR (Therapy)) OR (Therapies)) OR (Treatment)) OR (Treatments))) OR ("Disease Management"[Mesh])) OR (((Disease Management) OR (Management, Disease)) OR (Management, Disease)))

Embase-search

#1 ’management’/exp

#2 ’institutional management teams’ OR ’management audit’ OR ’office management’ OR ’pharmacy administration’ OR ’policy making’ OR ’practice management’ OR ’practice management, dental’ OR ’practice management, medical’ OR ’practice management, veterinary’ OR ’practice valuation and purchase’ OR ’management’

#3 ’therapy’/exp

#4 ’combination therapy’ OR ’disease therapy’ OR ’disease treatment’ OR ’diseases treatment’ OR ’disorder treatment’ OR ’disorders treatment’ OR ’efficacy, therapeutic’ OR ’illness treatment’ OR ’medical therapy’ OR ’medical treatment’ OR ’multiple therapy’ OR ’polytherapy’ OR ’somatotherapy’ OR ’therapeutic action’ OR ’therapeutic efficacy’ OR ’therapeutic trial’ OR ’therapeutic trials’ OR ’therapeutics’ OR ’therapy, medical’ OR ’treatment effectiveness’ OR ’treatment efficacy’ OR ’treatment, medical’ OR ’therapy’

#5 ’surgery’/exp

#6 ’diagnosis, surgical’ OR ’diagnostic techniques, surgical’ OR ’operation’ OR ’operation care’ OR ’operative intervention’ OR ’operative repair’ OR ’operative restoration’ OR ’operative surgery’ OR ’operative surgical procedure’ OR ’operative surgical procedures’ OR ’operative treatment’ OR ’research surgery’ OR ’resection’ OR ’specialties, surgical’ OR ’surgery, operative’ OR ’surgical care’ OR ’surgical correction’ OR ’surgical diagnosis’ OR ’surgical diagnostic techniques’ OR ’surgical exposure’ OR ’surgical intervention’ OR ’surgical management’ OR ’surgical operation’ OR ’surgical practice’ OR ’surgical procedures, operative’ OR ’surgical repair’ OR ’surgical research’ OR ’surgical restoration’ OR ’surgical service’ OR ’surgical speciality’ OR ’surgical specialties’ OR ’surgical specialty’ OR ’surgical therapy’ OR ’surgical treatment’ OR ’surgery’

#7 #1 OR #2 OR #3 OR #4 OR #5 OR #6

#8 ’sigmoid volvulus’

#9 #7 AND #8

The search strategy was formulated after consulting professional doctors and searchers. We conducted the literature search using search strategies developed by personnel trained in research methodology within our team. While no dedicated medical librarian was involved, all team members responsible for information retrieval received relevant training. Additionally, queries regarding evidence-based practices were directed to a designated individual in our department specializing in this area.

### Study records

#### Data management

All the retrieved literature data are imported into the software EndNote (version 21), and the process of literature screening and collation is carried out with this software. Before the formal literature screening, the participants are instructed how to operate software and master screening skills to ensure the authenticity, accuracy and comprehensiveness of the screening results.

#### Selection process

According to the inclusion and exclusion criteria described above, the two authors systematically analyze the title and abstract and read the full text for further literature content evaluation. Controversial literature is discussed with the third author to decide whether to be included. The overall screening process will be presented in the form of a flow chart.

#### Data collection process

The information extraction content of the literature generally includes three aspects. The first is basic information, such as the first author’s name, publication date, literature type, time and area of study implementation and number of participants. The second is the patient’s demographic characteristics, including the patient’s age, sex, race, ASA grade, and comorbidities. The last part includes data related to the operation, including the operation method, operation duration, presence of intestinal gangrene, intraoperative blood loss, postoperative hospital stay, postoperative complications, postoperative recurrence rate, postoperative mortality, causes of death and follow-up duration.

Similar to the literature screening process, the data extraction process is also independently completed by two authors, and any disagreements are resolved by another author. For missing data in the literature, we try to contact the author to obtain the original information. Multiple publications with the same data should also be checked and excluded. We will give priority to the latest research results for the data in the same region. For studies within the same time, data from studies with the largest sample sizes are extracted.

#### Risk of bias of individual studies

To facilitate the assessment of possible risk of bias for randomized controlled trials, two authors collect information by using the Cochrane Risk of Bias Tool [17]. This tool mainly evaluates bias risk from six fields and projects and is composed of selection bias, performance bias, detection bias, attrition bias, reporting bias, and other bias. Each indicator was judged using "low bias risk", "uncertain bias risk", and "high bias risk" (S2 Checklist). For the quality evaluation of nonrandomized controlled studies, we chose the Newcastle‒Ottawa Scale [18]. This table represents the score in the form of stars. The full score is 9 stars, and 5–9 stars indicate relatively high-quality articles. The literature is scored from three aspects: object selection, comparability, outcome and exposure. Each aspect has several evaluation items, and when the items meet the requirements, they are represented by one star (S3 Checklist). In cases of disagreement, the third examiner was the arbitrator.

## Data synthesis

### Measures of effect

We will synthesize the data from the included studies based on the type of intervention, population characteristics, and type of intervention outcome.

Relative risks are used to describe postoperative complications and additional interventions. Given the time-to-event nature of mortality, odds ratios are regarded as a preferred choice. Pooled quality-of-life statistics will be calculated as the mean differences ± standard deviations. Meanwhile, 95% confidence intervals and two-sided P values for each outcome were calculated. The threshold of statistical significance will be set at p <0.05. These summary statistics are combined and weighted by the number of patients in each study, and a forest plot is generated displaying the individual relative risks and weights as well as the pooled risk.

### Assessment of heterogeneity

We use the *I*^*2*^ test to evaluate the heterogeneity of the studies [19]. The *I*^*2*^ cutoff values corresponding to low, moderate and high heterogeneity were 25%, 50%, and 75%, respectively. Assuming that the results of the heterogeneity test of various studies are greater than 50%, the random effect model is used; if not, the pooled effect size is calculated by the fixed effect model. Subgroup analysis will explore study design and patient characteristics to identify sources of heterogeneity.

To improve the reliability of the research results, we adopted sensitivity analysis. One study is removed, and the remaining studies are combined for meta-analysis. By observing the changes in the combined results, we evaluated whether the original meta-analysis results significantly changed due to the influence of some studies.

Studies with potential bias will be excluded during sensitivity analysis. If the eliminated articles have an impact on the conclusion, differences between research design and implementation will be explored due to shed light on the specific factors influencing heterogeneity.

When the total number of included studies exceeded 10, Egger’s linear regression and funnel plot analysis were carried out to detect publication bias [20]. A significant publication was defined as a deviation of the intercept of Egger’s regression line from zero and a p value < 0.05. All data analysis processes will be carried out via R software (Version:4.3.3).

If there is great heterogeneity among studies, we will abandon the idea of meta-analysis and make a narrative and qualitative summary, that is, a systematic review or traditional review.

If the necessary data are available, subgroup analyses will be performed for people with an ASA<3 and an ASA≥3. Within each subgroup, and overall, we also plan to perform a subgroup analysis for the presence or absence of intestinal gangrene, endemic regions vs. non-endemic areas, whether laparoscopic surgeries are implemented.

### Confidence in the cumulative estimate

The quality of evidence for all outcomes will be judged using the Grading of Recommendations Assessment, Development and Evaluation working group methodology [21]. The quality of evidence will be expressed as high, medium, low or very low.

## Discussion

Generally speaking, the standard management of SV includes ameliorating manifestations, eliminating cause and preventing recurrence [22]. Given the nature of acute onset, patients are more concerned about the two former aims, in other words, they tend to reject prophylactic surgery after symptoms disappear [23]. Therefore, during practical clinical scenarios, the high recurrence rate are developed from a lot of reasons, although previous study recommended that resection surgery are acceptable, especially at the first admission. Results from a single-center, retrospective study from 2022 support a more aggressive surgical approach for such patients. In their study, the average number of hospital admissions was 2.18 and 4.43 for all patients and patients without surgery, respectively, and the ASA grade of patients generally increased with the increase in the number of hospital admissions [24]. The realization of decreased recurrence rate is needed to be further investigated.

With advancements in endoscopic technology and growing clinical experience, the management of endoscopic SV treatment has expanded beyond mere detorsion. PEC does not require general anesthesia and can be applied under endoscopic pre-administration and local anesthesia, providing detorsion treatment with less trauma to the intestines, which may lead to a shorter hospital stay and a faster post-operative recovery process [25]. Some scholars advocate for percutaneous endoscopic colopexy or colostomy (PEC) as alternatives to surgery for frail, elderly patients prone to relapses, who may not tolerate surgical interventions well due to their medical conditions [26, 27]. However, we chose not to include these approaches in our study design due to ongoing controversies regarding their effectiveness and safety. PEC has many shortcomings that need to be further overcome, such as: easy to damage adjacent blood vessels and intestines, easy to slip out when the location is inappropriate, and easy to induce in-situ infection [28]. Existing studies often suffer from small sample sizes and primarily consist of case reports, leading to inconsistent prognostic outcomes across different investigations. In one of the largest sample studies ever reported, the 30-day postoperative complications were 28% and the 90-day mortality was 18.5%, both at relatively high levels. Therefore, we believe that the major risks associated with PEC should be carefully weighted in actual clinical scenarios [29]. We look forward to future studies with larger sample sizes that may provide more definitive insights into the efficacy of these technologies.

The predominant advantage of this study is that we provide a comprehensively about the prognosis of almost all of the surgery underwent by the patients. which contributes to the clinical decision making and individualized therapy mode. Based on the scientific analysis process, we carried out this study by strict and we believe it has the potential to provide more reliable advice for current management of recurrent SV. In addition, we also plan to compare and demonstrate significant improvements in recurrence with specific surgical treatments by providing compelling data, not only for the doctors, but also for the patients.

Of course, we have to admit that there are some backwards for this present protocol. First, When assessing quality of the specific trials enrolled, we found them lacked in sufficient sample and belonged to retrospective observational study. Meanwhile, definition of outcomes, diagnosis methods, data analysis, form of the statistics and other factors vary from different articles, resulting to the increased heterogeneity. In addition, we understand that removing samples less than 10 may have implications for some rare surgeries or procedures, such as PEC. However, this decision was made to ensure the scientific rigor and reliability of the research. By focusing on studies with larger sample sizes, we are able to analyze and extrapolate conclusions more reliably, improving the quality and reliability of our studies.

We anticipate observing a significant difference between resection and non-resection surgery in preventing SV recurrence. From which, we can shed a light on the better algorithm for SV and provide a direction to addressing treatment dilemma.

## Supporting information

S1 Checklist PRISMA-P (Preferred Reporting Items for Systematic review and Meta-Analysis Protocols) 2015 checklist: Recommended items to address in a systematic review protocol*. (DOCX)

S2 Checklist Risk of bias evaluation-Randomized controlled trials. (DOCX)

S3 Checklist Risk of bias evaluation-Observational study. (DOCX)
