# Supplementary material for: Comparison of the ability of resection versus nonresection surgery to prevent the recurrence of sigmoid volvulus: A protocol of a meta-analysis and systematic review
Source: PLoS One. 2024 Sep 24;19(9):e0310402. doi: 10.1371/journal.pone.0310402 (PMC11421782; doi:10.1371/journal.pone.0310402)
Supplement: S2 Checklist — (DOCX) [file pone.0310402.s002.docx]

**Supporting file1: Risk of bias evaluation-Randomized controlled trials**

**Manuscript: Comparison of the ability of resection versus nonresection surgery to prevent the recurrence of sigmoid volvulus: a protocol of a meta-analysis and systematic review**

**Table1 Cochrane Collaboration’s tool for assessing risk of bias**

| Selection bias | Random sequence generation | Describe the method used to generate the allocation sequence in sufficient detail to allow an assessment of whether it should produce comparable groups | Selection bias (biased allocation to interventions) due to inadequate generation of a randomised sequence |
| --- | --- | --- | --- |
|  | Allocation concealment | Describe the method used to conceal the allocation sequence in sufficient detail to determine whether intervention allocations could have been foreseen before or during enrolment | Selection bias (biased allocation to interventions) due to inadequate concealment of allocations before assignment |
| Performance bias | Blinding of participants and personnel* | Describe all measures used, if any, to blind trial participants and researchers from knowledge of which intervention a participant received. Provide any information relating to whether the intended blinding was effective | Performance bias due to knowledge of the allocated interventions by participants and personnel during the study |
| Detection bias | Blinding of outcome assessment* | Describe all measures used, if any, to blind outcome assessment from knowledge of which intervention a participant received. Provide any information relating to whether the intended blinding was effective | Detection bias due to knowledge of the allocated interventions by outcome assessment |
| Attrition bias | Incomplete outcome data* | Describe the completeness of outcome data for each main outcome, including attrition and exclusions from the analysis. State whether attrition and exclusions were reported, the numbers in each intervention group (compared with total randomised participants), reasons for attrition or exclusions where reported, and any reinclusions in analyses for the review | Attrition bias due to amount, nature, or handling of incomplete outcome data |
| Reporting bias | Selective reporting | State how selective outcome reporting was examined and what was found | Reporting bias due to selective outcome reporting |
| Other bias | Anything else, ideally prespecified | State any important concerns about bias not covered in the other domains in the tool | Bias due to problems not covered elsewhere |

*Assessments should be made for each main outcome or class of outcomes.

*From:Higgins JPT, Altman DG, Gøtzsche PC, Jüni P, Moher D, Oxman AD, et al. The Cochrane Collaboration's tool for assessing risk of bias in randomised trials. BMJ (Clinical research ed). 2011;343:d5928. doi: 10.1136/bmj.d5928. PubMed PMID: 22008217; PubMed Central PMCID: Q1.*
